# Surgical excision of post-traumatic myositis ossificans of the adductor longus in a football player

**DOI:** 10.1136/bcr-2019-233504

**Published:** 2020-03-03

**Authors:** Gijs Herman Joseph de Smet, Steven E Buijk, Adam Weir

**Affiliations:** 1 Department of Surgery, Erasmus Medical Center, Rotterdam, Zuid-Holland, The Netherlands; 2 Department of Surgery, IJsselland Ziekenhuis, Capelle aan den IJssel, Zuid-Holland, The Netherlands; 3 Department of Orthopaedics, Erasmus Medical Center, Rotterdam, Zuid-Holland, The Netherlands

**Keywords:** trauma, sports and exercise medicine, surgery

## Abstract

A football player was diagnosed with myositis ossificans of his right adductor longus muscle after an acute injury. Conservative treatment failed and 1 year after the initial trauma the patient underwent surgical excision of a large ossification. Seven months postoperatively, the patient was fully recovered and returned to his preinjury activity levels. We present our approach to this case and discuss our considerations, referring to background information about this rare disease.

## Background

Myositis ossificans (MO) is a non-neoplastic heterotopic ossification that develops within skeletal muscle or soft tissue. The exact pathophysiological pathway of this rare disease is still not fully understood. It is believed that, in response to tissue injury and subsequent inflammation, stem cells are dysregulated and stimulated to follow osteogenic pathways, resulting in ossification.^1^ The disorder can be differentiated into four subtypes: (1) MO traumatica; (2) MO progressiva (hereditary form); (3) MO associated with paraplegia and (4) MO associated with burns.^2 3^ Myositis ossificans traumatica (MOT) is the most common type and accounts for 60%–75% of all cases.^4 5^ MOT usually occurs in the girdles and limbs of active young men after major or repeated minor trauma. The brachialis, quadriceps and adductor group muscles are more prone to MOT according to the literature.^6 7^

MOT can be mistaken for malignant lesions such as osteosarcoma or rhabdomyosarcoma. Therefore, appropriate diagnostic tests are of paramount importance. However, the stage of the disease affects its presentation and might lead to difficulties in the interpretation of radiological and histological findings.^8 9^

As MOT is self-limiting and self-resolving in most cases, conservative treatment with rest, adequate pain relief, and restoring function and range of motion is often effective for returning to sports and activities.^8 10^ Other non-surgical options include aspirating hematomas, non-steroidal anti-inflammatory drugs (NSAIDs) and extracorporeal shockwave therapy (ESWT). Surgical intervention should be considered when the consolidated mass persists and causes symptoms.^11^

We present the first reported case of MOT of the adductor longus muscle in a football player, which was treated successfully with surgical excision.

## Case presentation

A 23-year-old male football player, without any relevant medical history, was referred by the general practitioner to our outpatient clinic with complaints of the right groin. One year earlier, during intensive training, the patient felt acute intense pain in the right groin twice when kicking the ball. After training, he felt swelling and pain in the right adductor region. After a period of rest, physiotherapy was started. Low-intensity exercises were gradually performed and he started cycling. He tried to resume his football training sessions. Unfortunately, the pain in his groin recurred. He repeated physical rehabilitation from the beginning three times. However, the intensity of the pain increased over time and began to also limit his activities of daily living.

On physical examination, palpation of the origin of the adductor muscles at the right pubic bone was painful and revealed a small gap in the tendon located just distal to the insertion. Distal to the gap, a very firm mass of about 7 cm long and 1 cm wide could be palpated. It felt as if a short pencil was lying under the skin. Palpation of the mass was extremely painful. The right adductor muscle was shortened and resisted adductor muscle strength testing was weak and painful. Further examination showed no signs of nerve entrapment with normal sensation of the skin. Clinical examination of the hip joint range of motion was pain free.

## Investigations

Ultrasound imaging showed an old rupture of the right adductor longus muscle with a small fluid collection located proximally. Distally, a calcification of 8 cm was seen, indicating a chronic disease process with signs of active inflammation.

**Figure 1:**
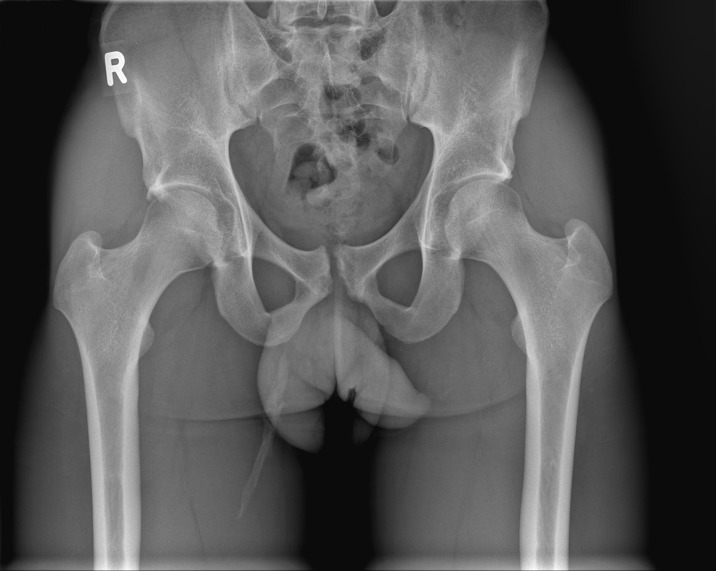
Pelvic X-ray showing calcification in the adductor region.

An X-ray clearly showed a heterotopic ossification of the right adductor longus muscle (figure 1).

## Differential diagnosis

The differential diagnosis of MOT includes benign and malignant diseases. Benign diseases that may be confused with MOT are local abscess formation, a calcified fibrous tumour and periosteal reaction, whereas osteosarcoma and rhabdomyosarcoma are important malignant diseases to consider.^9^ In the present case, it was possible to distinguish MOT from all these other diagnoses through the combination of the specific history of a sports trauma, the physical examination, combined with both ultrasound and X-ray imaging.

## Treatment

**Figure 2:**
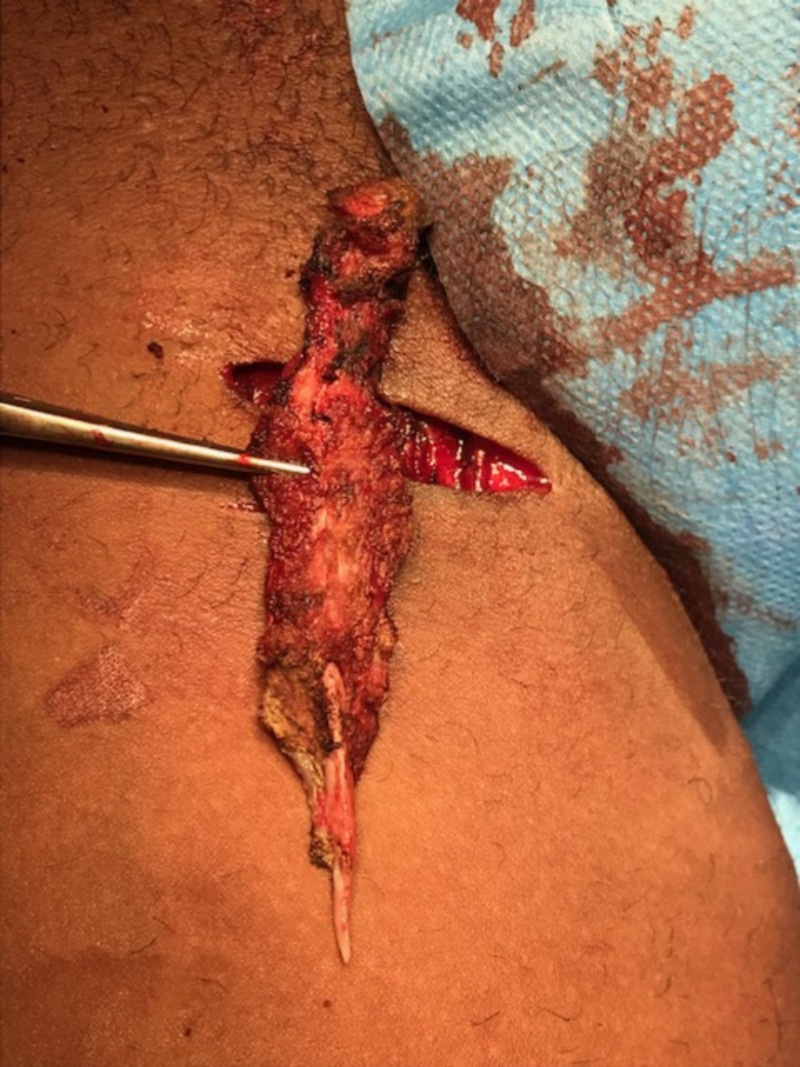
Peroperative photo of resected ossified mass of the right adductor longus muscle.

**Figure 3:**
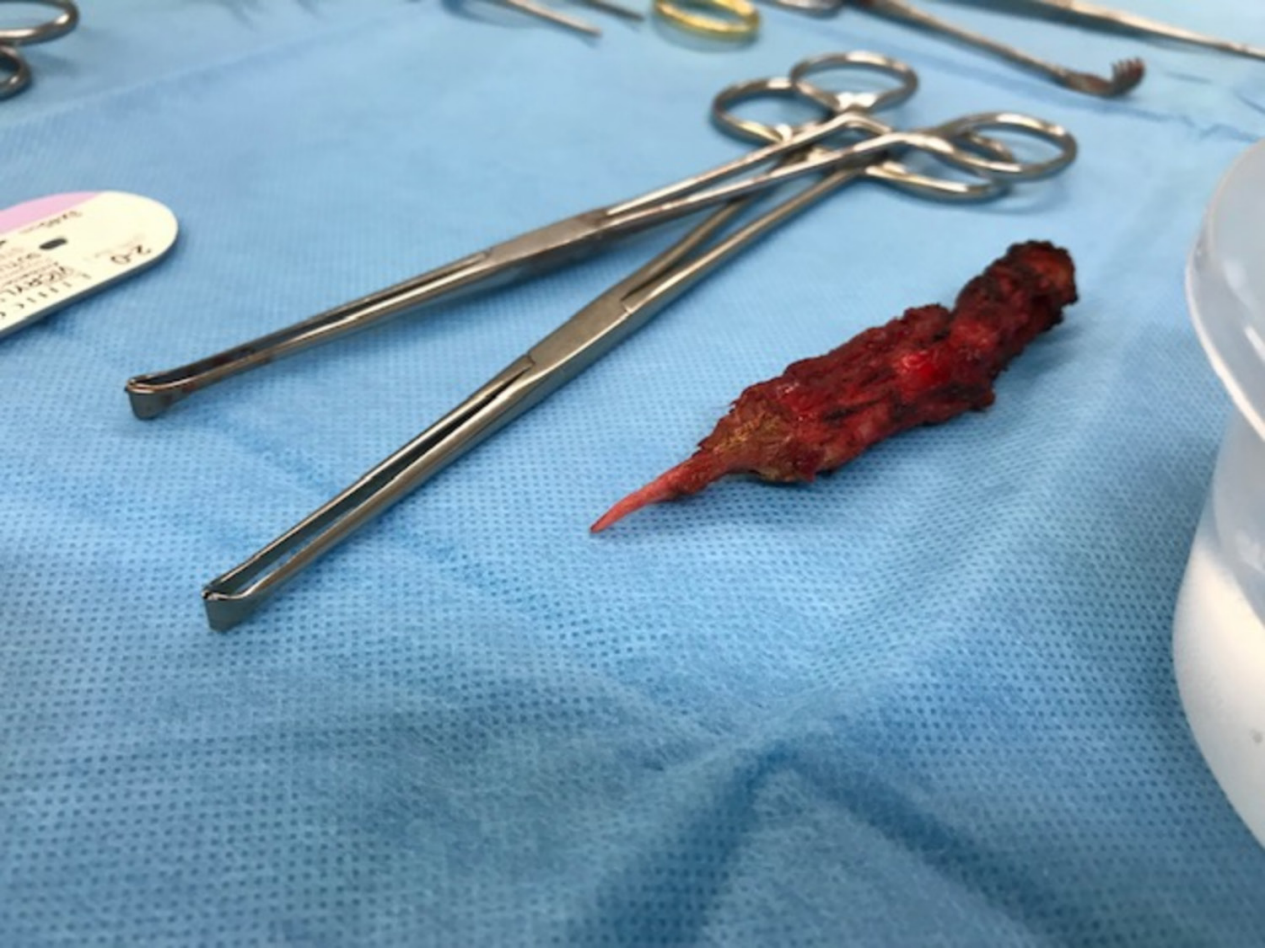
Resected ossification along surgical instruments.

Surgical excision of the MOT was performed. Incision was made in the direction of Langer’s lines at the level of the palpable mass. Subsequently, the great saphenous vein was ligated. After identification of the mass, it was peeled off the adductor longus muscle in one piece (figures 2 and 3). The muscle belly and adductor longus fascia were sutured with Vicryl and the skin was closed intracutaneously with Monocryl.

## Outcome and follow-up

Histopathological analysis showed a round nodulus consisting of bone tissue with bone trabeculae surrounded with osteoblast cells. No typical zones could be recognised and the centre of the nodulus did not contain any cells.

At 3 months of follow-up, the patient was free of symptoms and followed the rehabilitation programme at his local football club. Exercises were intensified using weights, as well as with cycling, running and resuming light training sessions. No abnormalities were found on physical examination of the adductors on palpation, stretching and resistance testing. Dynamometry showed adductor strength of 157 N on the right (R) and of 177 N on the left (L), add abductor strength as this is needed to calculate with adductor/abductor ratio of 1.03 R and 1.32 L.

At 5 months of follow-up, the patient, although not at full intensity, participated in football training sessions three times a week. Dynamometry showed adductor strength R of 181 N and L of 176 N, with adductor/abductor ratio of 1.16 R and 1.17 L.

**Figure 4:**
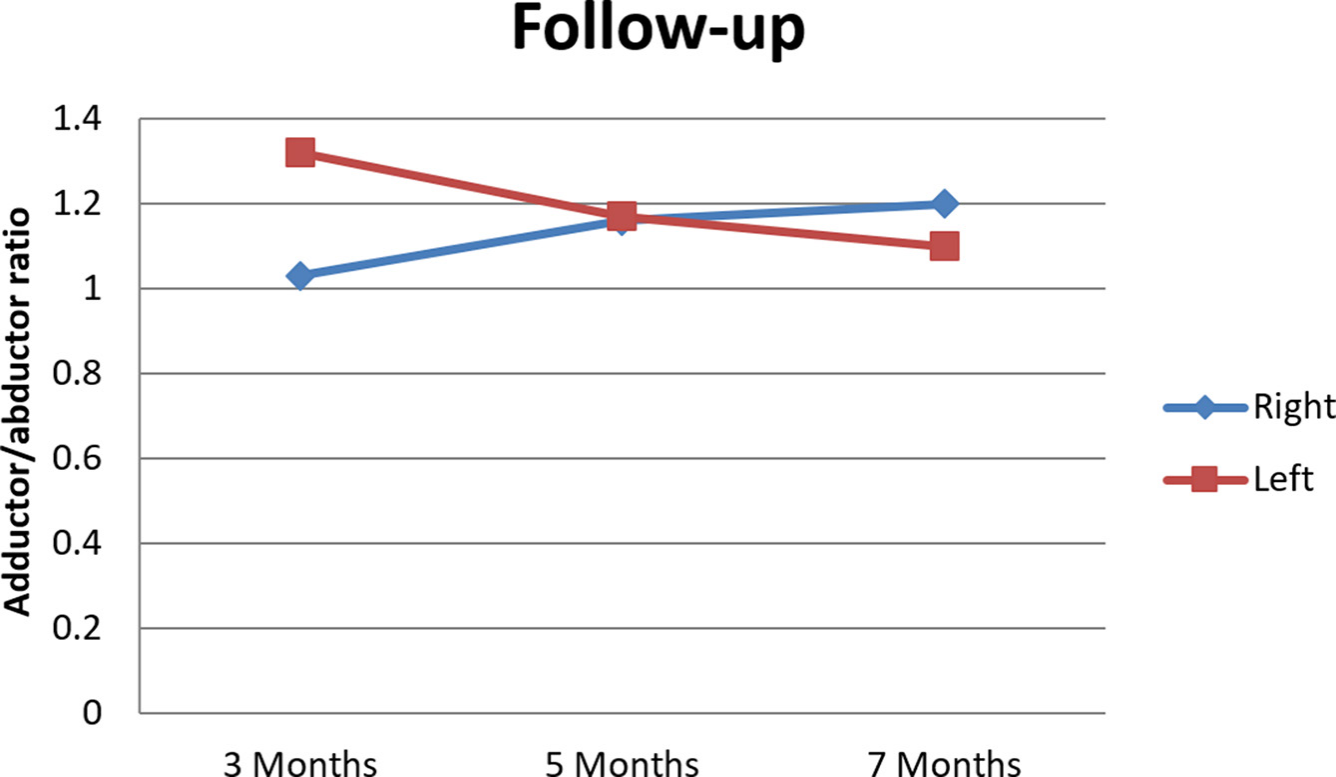
Follow-up adductor/abductor ratio.

After 7 months, the patient was fully recovered and participated in football matches without any pain. Dynamometry showed adductor strength R of 213 N and L of 190 N, with adductor/abductor ratio of 1.20 R and 1.10 L. All dynamometry outcomes collected during the follow-up period are presented in table 1 and figure 4.

**Table 1:** Follow-up dynamometry outcomes

Dynamotry (in N)	Adductor muscles		Abductor muscles		Adductor/abductor ratio	
Follow-up postoperative	Right	Left	Right	Left	Right	Left
3 Months	157	177	152	134	1.03	1.32
5 Months	181	176	156	151	1.16	1.17
7 Months	213	190	178	173	1.20	1.10

## Discussion

MOT of the adductor muscles in athletes has been described previously.^11–13^ However, to the best of our knowledge, this is the first case reported in the literature of MOT of the adductor longus muscle. In football players, MOT is often the result of direct contusion of the adductor muscles or eccentric muscle overload that leads to an acute muscle/tendon injury.^11^ The latter was the trauma mechanism in the present case. Muscle tears can cause intermuscular and intramuscular hematoma formation, which is known to be an important event in the cascade of MOT.^6^ To minimise hematoma formation at the injury site, first aid following the rest, ice, compression and elevation principle is recommended.^14^ In this case, it is not clear whether the patient has followed this therapy sufficiently to prevent the development of MOT. Local inflammation and macrophage accumulation are also crucial steps in the cascade of heterotopic ossification formation, stimulating osteogenic factors, including bone morphogenetic proteins.^1^ Hence, NSAID is proposed to prevent MOT in an early phase.^15^ On the other hand, NSAIDs block the cyclooxygenase-2 pathway which is an essential pathway for skeletal muscle repair and muscle growth.^16 17^ The effect of the preventive use of NSAIDs in the early phase of MOT is controversial and warrants further investigation. In this case, NSAIDs were prescribed in the late phase as they had not yet been used and we felt it was worth trying both to reduce the pain and to try and prevent further formation. We also considered starting with ESWT. Due to the relatively large size of the calcification, we expected that the ESWT would take many sessions and be very painful. After discussing this with the patient, he decided not to try this.

Surgical excision of MOT can be considered when pain persists, if muscle weakness is present, or in case of a limited range of motion.^10^ It is crucial that surgical excision of MOT is planned to take place at least 6 months after the initial trauma, when the ossification is fully matured.^11^ Excision of immature ossifications often leads to local recurrence.^6^ Our patient met all the above-mentioned conditions for surgical excision: increased pain, functional impairment and failure to resume sport activities for over 1 year.

Imaging can be useful to diagnose MOT correctly. Especially when considering that malignant diseases such as osteosarcoma and rhabdomyosarcoma can have similar clinical presentations.^9^ Still, the different imaging modalities have to be interpreted with caution, since radiological findings depend on the stage of the disease.^8 9^ In combination with a thorough patient history and physical examination, MOT can be distinguished adequately from other diseases. Both Devilbiss *et al* 8 and Li *et al* 18 provide a clear overview of specific characteristics of MO which could be helpful to differentiate between diagnoses.

Guideline recommendations on return to sports for athletes with MO treated conservatively are lacking, let alone for athletes after surgical excision. Orava *et al* 11 allowed athletes to return to sports 4–6 weeks after surgical excision of MOT. For athletes with persisting symptoms and those who did not fulfil the return to sport criteria, this period was extended for up to another month. In their series, 30 of the 32 athletes were able to resume preinjury Tegner activity levels.^11^ In our case, we decided to refer the patient to the well-known physiotherapists of the local football club for postoperative rehabilitation, closely following up the patient with outpatient visits at 4 weeks, 3 months, 5 months and 7 months. Seven months postoperatively, the patient had returned to preinjury activity levels and resumed football matches.

To the best of our knowledge, this is the first case of MOT of the adductor longus muscle described in the literature. This case emphasises that surgical excision of MOT of the adductor longus muscle can be an effective option when conservative treatment options have failed.

**Patient’s perspective**

After the last follow-up moment, we asked the patient to describe the clinical process, including the surgical treatment on his perspective. He described this as follows: at first, I was a bit shocked when I was told that MOT of the adductor longus muscle is a rare disease and that it was never seen by the sports doctor. At the same time, I trusted the sports doctor, thanks to his open and professional approach. I was informed about the potential risk in losing some of the muscle strength of my right leg when choosing to undergo surgery. However, surgery was the obvious choice for me because the MOT affected my activities of daily living. After surgery, the pain was slightly worse than before but this reduced quickly and I could resume my activities of daily living. Furthermore, due to the close follow-up and the rehabilitation programme, I experience no (pain) complaints anymore during football and I am able to fully participate in football matches. I am very satisfied with the treatment and the end result.

**Learning points**

- Myositis ossificans traumatica (MOT) of the adductor muscles is extremely rare.
- Immediate treatment of a skeletal muscle injury with the rest, ice, compression and elevation principle might help in the prevention of MOT.
- Surgical excision of MOT of the adductor longus muscle is a good option when conservative treatments fail.
